# Prediction of internal jugular vein catheter length inserted through the posterior approach of the sternocleidomastoid muscle

**DOI:** 10.1097/MD.0000000000038876

**Published:** 2024-07-26

**Authors:** Qunxiang Chen, Xiaoyu Zhang, Huanlin Zhang, Jie Li, Yan Zhang, Kaixiang Zhang, Xi Chen

**Affiliations:** a Department of Oncology, The 900th Hospital of Joint Logistic Support Force, PLA, Fuzhou, China; b Fuzong Clinical Medical College of Fujian Medical University, Fuzhou, China.

**Keywords:** body height, catheter length, chest length, infusion port, regression analysis

## Abstract

This study aimed to determine an equation to estimate the optimal insertion length for catheter placement via the posterior approach of the sternocleidomastoid muscle in cancer patients. This retrospective study included patients with cancer who underwent infusion port implantation surgery in the Oncology Department of the 900th Hospital of Joint Logistic Support Force of the Chinese People Liberation Army from April 2017 to September 2023. Patient height (H), weight (W), chest length (C), and length of the internal jugular vein catheter (L) were collected from medical records. The patients were randomized 7:3 to the training and validation sets. Linear regression analyses were used in the training set to determine formulas to predict catheter length. The formula predictive value was analyzed using the Bland-Altman method in the validation set. This study included 336 patients, with a mean age of 58.27 ± 11.70 years, randomized in the training (n = 235) and validation (n = 101) sets. Linear regression analysis revealed that the equations for catheter length relative to H, body mass index (BMI), and C are L = 0.144 × H - 8.258 (R² = 0.608, *P* < .001), L = −0.103 × B + 17.384 (R² = 0.055, *P* < .001), and L = 0.477 × C + 1.769 (R² = 0.342, *P* < .001), respectively. The multivariable linear regression analysis showed that the equation between the length of the catheter and H and C was L = 0.131 × H + 0.086 × C-8.515 (R² = 0.614, *P* < .001). The Bland-Altman analysis in the validation set showed that the predicted values of internal jugular vein catheter length and the actual values showed good agreement. The optimal L might be determined by simple formulas based on patients H and C.

## 1. Introduction

Infusion ports are widely used for venous chemotherapy, nutritional support, etc, providing great convenience for patients with advanced tumors by avoiding repeated venipuncture.^[1]^ The subclavian and internal jugular veins are the most used puncture vessels for infusion port implantation.^[2]^

Internal jugular vein catheterization is divided into the middle and posterior approaches according to the relative position between the puncture point and the sternocleidomastoid muscle.^[3,4]^ The middle approach puncture point is located between the sternal and clavicular branches of the sternocleidomastoid muscle, while the posterior approach puncture point is located behind the clavicular branch of the sternocleidomastoid muscle,^[3,4]^ referred to in this paper as the posterior approach of the sternocleidomastoid muscle. Nevertheless, regardless of the chosen approach, it is essential to ensure that the tip of the catheter does not exceed the junction of the superior vena cava and the right atrium, making accurate preoperative estimation crucial.^[3–6]^ Accurate catheter length estimation is expected to avoid the psychological impact on patient and infection risks secondary to catheter adjustment, especially for infusion ports.

There are no gold standard methods for estimating the insertion depth of catheters. Some clinicians have derived estimation formulas for catheter length using statistical methods, which can serve as references. The Peres equation, (height [H]/10) (in cm), was formulated in 1990.^[7]^ The Czepizak formula (H/10)-1 (in cm), was formulated in 1995 as an improvement of the Peres formula.^[8]^ Both formulas were designed for internal jugular vein catheterization in general, without concerns regarding the approach or anthropometric features. Nevertheless, there is currently no literature reporting the estimation of catheter length specifically for implantation via the posterior approach of the sternocleidomastoid muscle.

Therefore, this study aimed to derive an estimation equation to estimate the optimal insertion length for catheter placement via the posterior approach of the sternocleidomastoid muscle in cancer patients using the body surface parameters.

## 2. Methods

### 2.1. Study design and patients

This retrospective study included patients with cancer who underwent infusion port implantation surgery in the Oncology Department of the 900th Hospital of Joint Logistic Support Force of the Chinese People Liberation Army from April 2017 to September 2023. The study was approved by the ethics committee of the 900th Hospital of Joint Logistic Support Force of the Chinese People Liberation Army. The requirement for individual informed consent was waived by the committee due to the retrospective nature of the study.

**Figure 1.**
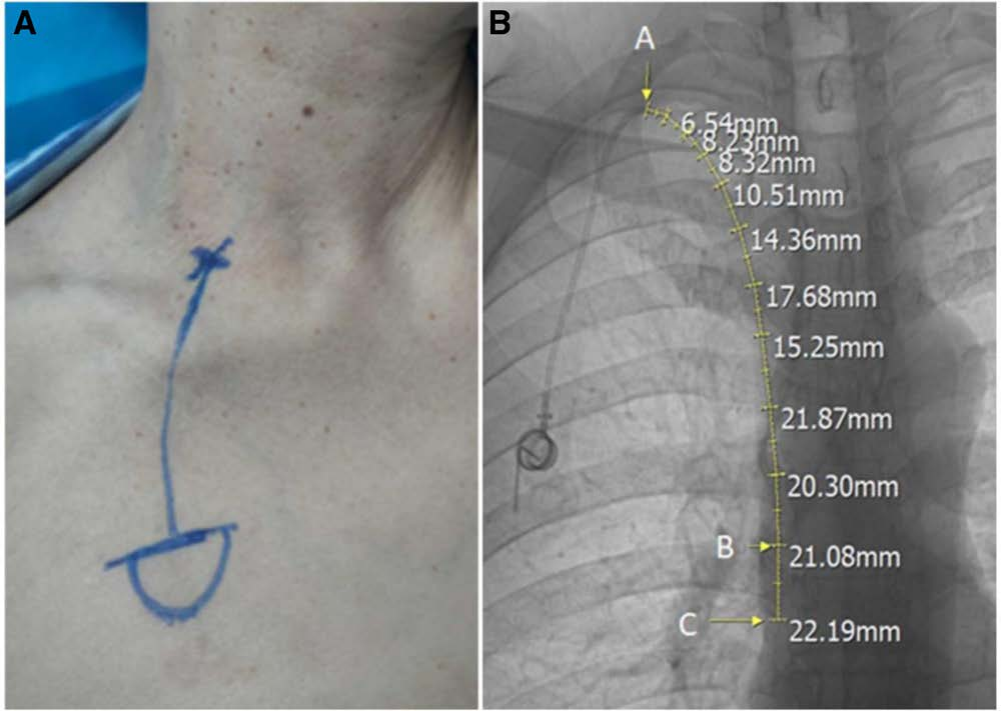
(A) Placement of the infusion port via the posterior approach of the sternocleidomastoid muscle (female, 59 yr old, diagnosed with colon cancer with multiple metastases throughout the body). (B) Measurement of the length of the internal jugular vein catheter. Point A indicates the entry point of the catheter into the internal jugular vein, point B indicates the position of the catheter tip, and point C indicates the junction of the superior vena cava and the right atrium. The distance from point A to point B represents the actual length of the internal jugular vein catheter, while the distance from point B to point C represents the extended length.

The inclusion criteria were ≥18 years of age and placement of the catheter from the posterior aspect of the clavicular branch of the right sternocleidomastoid muscle, specifically selecting the posterior approach of the sternocleidomastoid muscle (Fig. 1A). The exclusion criteria were the presence of chest or thoracic vertebral deformities, such as mediastinal deviation due to pleural effusion, lung destruction, or thoracic scoliosis, or the inability to determine accurately the tip of the catheter on postoperative chest X-rays in the anteroposterior view.

### 2.2. Data collection

Patient H, weight (W), chest length (C), and length of the internal jugular vein catheter (L) were collected from the patient medical records. Body mass index (BMI) was calculated as BMI = W (kg)/H (m)^2^. C referred to the length of the thoracic trunk segment, obtained by measuring the vertical H of the thoracic vertebrae on the patient CT scan. The internal jugular vein catheter length was measured on postoperative anteroposterior chest X-rays (Fig. 1B). It started from the highest point of the arc of internal jugular vein catheter in the neck and extended downward along the catheter to the level of the junction of the superior vena cava and the right atrium, including the extended length.^[9]^ Two attending physicians jointly scrutinized the patients included in the research and measured each set of data respectively, and afterward, the average values were taken.

### 2.3. Statistical analysis

Statistical analysis was performed using SPSS version 20.0 (IBM Corp. Armonk, NY). The continuous variables conforming to the normal distribution were presented as means ± standard deviations and analyzed using Student *t* test. The categorical variables were described as n (%) and analyzed using the chi-squared test. All patients were randomly divided into the training and validation sets at a ratio of 7:3. The training set was used to establish the predictive formula, and the validation set was used to evaluate the predictive performance of the formula. The derivation of the formula in the training set was based on linear regression analyses of patient H, BMI, and C. Subsequently, the H, BMI, and C data from the validation set were substituted into the regression equation derived from the training set to calculate the predicted L. The Bland-Altman method was used for validation of prediction equation. Two-sided P-values < 0.05 indicated statistical significance.

## 3. Results

**Figure 2.**
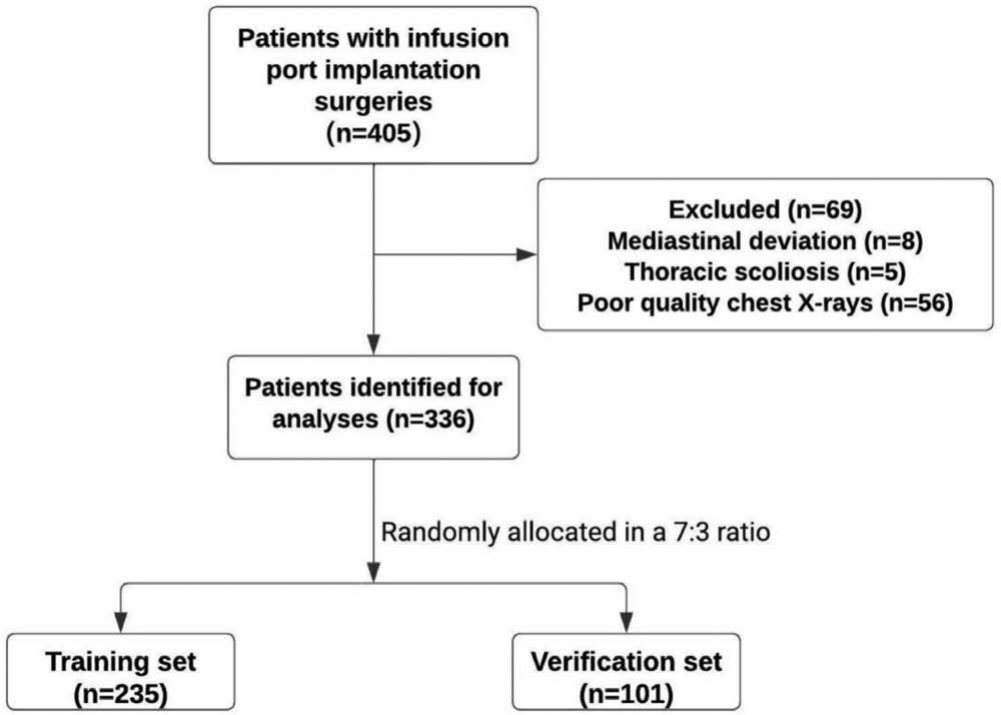
Patient inclusion flowchart.

From April 2017 to September 2023, 405 patients underwent port catheter implantation; 69 were excluded (8 for mediastinal deviation, 5 for thoracic scoliosis, and 56 for poor X-ray image quality), and 336 were included, randomized in the training (n = 235) and validation (n = 101) sets (Fig. 2).

Among the 336 patients, there were 165 males and 171 females, with an age range of 18 to 84 years and a mean age of 58.27 ± 11.70 years. The distribution of primary malignancies included 81 patients with colorectal cancer, 52 with lung cancer, 42 with gastric cancer, 39 with gynecological malignancies, 31 with breast cancer, 24 with pancreatic and biliary tract cancers, 13 with head and neck malignancies, 10 with urological cancer, 9 with esophageal cancer, 8 with soft tissue cancers, and 21 with other cancers. There were no significant differences between the 2 sets regarding age, sex, H, C, and cancer types (all *P* > .05), but the patients in the training set showed a higher W (60.43 ± 10.37 vs 57.72 ± 10.42, *P* = .029) and a higher BMI (23.07 ± 3.45 vs 21.87 ± 3.39, *P* = .040) than those in the validation set (Table 1).

**Table 1:** Characteristics of the patients.

	Total (n = 336)	Training set (n = 235)	Verification set (n = 101)	*P*
Age (yr)	58.27 ± 11.70	58.92 ± 11.63	56.76 ± 11.77	.121
Gender (n, %)				.887
Male	165 (49.11%)	116 (49.36%)	49 (48.51%)	
Female	171 (50.89%)	119 (50.64%)	52 (51.49%)	
Height (cm)	161.93 ± 8.45	161.75 ± 8.27	162.35 ± 8.89	.552
Weight (kg)	59.62 ± 10.45	60.43 ± 10.37	57.72 ± 10.42	.029
BMI (kg/m^2^)	22.71 ± 3.47	23.07 ± 3.45	21.87 ± 3.39	.04
Chest length (cm)	27.79 ± 1.87	27.72 ± 1.87	27.94 ± 1.86	.341
Primary tumor (n, %)				.388
Colorectal cancer	81 (24.11%)	52 (22.13%)	29 (28.71%)	
Lung cancer	52 (15.48%)	36 (15.32%)	16 (15.84%)	
Gastric cancer	42 (12.50%)	25 (10.64%)	17 (16.83%)	
Gynecological malignancies	39 (11.61%)	30 (12.77%)	9 (8.91%)	
Breast cancer	31 (9.23%)	25 (10.64%)	6 (5.94%)	
Pancreatic and biliary tract cancers	24 (7.14%)	15 (6.38%)	9 (8.91%)	
Head and neck malignancies	13 (3.87%)	9 (3.83%)	4 (3.96%)	
Urological cancer	10 (2.98%)	8 (3.40%)	2 (1.98%)	
Esophageal cancer	9 (2.68%)	7 (2.98%)	2 (1.98%)	
Soft tissue cancers	8 (2.38%)	5 (2.13%)	3 (2.97%)	
Others	21 (6.25%)	17 (7.23%)	4 (3.96%)	

BMI = body mass index.

The regression equation between the L and H was L = 0.144 × H-8.258. H significantly positively predicted the L (*P* < .001) and explained 60.8% of the variance in the length of the catheter. The regression equation between the length of the catheter and BMI was L = -0.103 × B + 17.384. BMI significantly negatively predicted the length of the catheter (*P* < .001) and explained 5.5% of the variance in the length of the catheter. The regression equation between the length of the catheter and C was L = 0.477 × C + 1.769. C significantly positively predicted the length of the catheter (*P* < .001). C explained 34.2% of the variance in the length of the catheter. The results of the multivariable linear regression analysis showed that the regression equation between the length of the catheter and H and C was L = 0.131 × H + 0.086 × C-8.515 (*P* < .001). Among them, H (β = 0.708, *P* < .05) significantly positively predicted the length of the catheter, while C (β = 0.106, *P* > .05) did not (Table 2, Figure S1, http://links.lww.com/MD/N221). The possible reason for that C showed significant prediction for the catheter length in the univariable analysis while not in the multivariable analysis might be that there was a collinearity relationship between C and H. Moreover, due to the fact that BMI explained the variance of the catheter length extremely feebly, it was not consequently included in the final multivariable model.

**Table 2:** Regression equations.

Indicator	Equation	R^2^	*P*
Height	L = 0.144 × H-8.258	0.608	<.001
BMI	L = −0.103 × B + 17.384	0.055	<.001
Chest length	L = 0.477 × C + 1.769	0.342	<.001
Height, chest length	L = 0.131 × H + 0.086 × C-8.515	0.614	<.001

BMI = body mass index.

**Figure 3.**
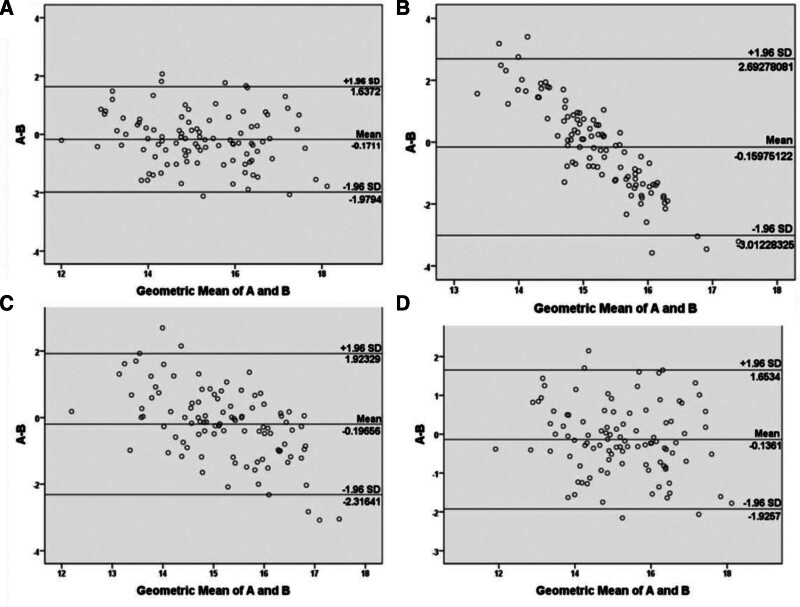
(A) A: length of the internal jugular vein catheter measured by L = 0.144 × H-8.258 (cm); B: length of the internal jugular vein catheter (cm). (B) A: length of the internal jugular vein catheter measured by L = −0.103 × B + 17.384 (cm); B: length of the internal jugular vein catheter (cm). (C) A: length of the internal jugular vein catheter measured by L = 0.477 × C + 1.769 (cm); B: length of the internal jugular vein catheter (cm). (D) A: length of the internal jugular vein catheter measured by L = 0.131 × H + 0.086 × C-8.515 (cm); B: length of the internal jugular vein catheter (cm).

Using the validation set, the results of the Bland-Altman analysis showed that the predicted values of internal jugular vein catheter length using the various equations and the actual values showed good agreement, with most of the points being observed between ± 1.96 standard deviations around the mean (Fig. 3).

## 4. Discussion

This study demonstrated that patient H combined with C might to be a practical measure for determining the optimal catheter length.

Infusion ports are widely used in cancer patients receiving intravenous systemic therapy or intravenous nutritional support, helping them avoid the pain of repeated peripheral venous punctures.^[10]^ Proficiency in implanting an infusion port is essential to minimize complications associated with vascular punctures and to ensure that the catheter length is appropriate and does no damage to the vena cava and right atrium.^[11,12]^ It also helps avoid potential infection risks associated with secondary adjustments to the catheter length and prevents patient discomfort, anxiety, and tension.^[12]^

To avoid catheters being too long or too short, many clinicians have experimented with various approaches.^[13,14]^ For example, digital subtraction angiography (DSA) guidance or intracavitary electrocardiogram techniques can be used during port implantation.^[15–18]^ These methods offer reliability as the main advantage. On the other hand, they require additional equipment, prolong surgical duration, and increase medical costs, imposing an additional financial burden on the patients.^[19]^ Moreover, performing surgery under DSA exposes the patients and medical staff to radiation. Some clinicians have also explored meaningful ways to improve catheter placement accuracy by measuring implanted catheter lengths and correlating them with anthropometric parameters such as H.^[7,8]^

In general, taller patients typically require longer internal jugular vein catheter lengths. Indeed, the original Peres^[7]^ and Czepizak^[8]^ formulas were solely based on H to determine the catheter length. Same finding was also reported in neonates.^[20]^Still, the Peres equation^[7]^ has been shown to have a lower performance in female and obese Turkish individuals.^[6]^ Struck et al^[21]^ showed that the Peres and Czepizak formulas performed adequately for individuals 170–180 cm tall but less well in smaller or taller individuals. In addition, those formulas have a 48%-94.5% correct placement rate.^[22,23]^ Therefore, using H as the sole variable for calculation of the catheter length is imprecise. Indeed, patients with a longer C relative to their H may still need a longer catheter, even if they are not particularly tall. Using linear regression analysis, the present study could determine that the L is linearly positively correlated with H and C, and negatively with BMI. Specifically, the regression equation derived was L = 0.131 × H + 0.086 × C-8.515 for H and C.

To further facilitate clinical application, software based on the regression equations mentioned above should be developed. Before surgery, clinicians can simply measure relevant parameters, such as patient H and C, and then input them into the software. It will enable the direct calculation of the recommended length for the internal jugular vein catheter. Still, other methods to determine catheter length were described and could be considered to optimize future equations. Methods based on body surface measurement,^[24,25]^ computed tomography measurements,^[26]^ and bifurcation techniques^[25]^ yielded good performance.

This study has limitations. Due to the relatively small number of patients in this study, there may still be some deviation between the predicted length of the catheter using the regression equations and the actual required length. This limitation should be considered when applying the regression equations, and adjustments should be made to the calculated catheter length based on individual clinical circumstances. The equations should also be validated in larger numbers of patients to refine them. All included patients were Chinese, and ethnic differences are possible.

In conclusion, this study showed that combining patient H with C could be a practical approach for determining optimal L. Future prospective studies are required to assess practical application of equation.

## Author contributions

**Conceptualization:** Qunxiang Chen, Xiaoyu Zhang, Huanlin Zhang, Jie Li.

**Data curation:** Qunxiang Chen, Xiaoyu Zhang, Huanlin Zhang, Jie Li, Yan Zhang, Kaixiang Zhang, Xi Chen.

**Formal analysis:** Qunxiang Chen, Xiaoyu Zhang, Huanlin Zhang, Jie Li, Yan Zhang, Kaixiang Zhang, Xi Chen.

**Investigation:** Qunxiang Chen, Yan Zhang, Kaixiang Zhang, Xi Chen.

**Methodology:** Qunxiang Chen.

**Writing – original draft:** Qunxiang Chen, Yan Zhang, Kaixiang Zhang, Xi Chen.

**Writing – review & editing:** Qunxiang Chen, Xiaoyu Zhang, Huanlin Zhang, Jie Li, Yan Zhang, Kaixiang Zhang, Xi Chen.

## Supplementary Material
