# Supplementary figures and images for: Prediction of internal jugular vein catheter length inserted through the posterior approach of the sternocleidomastoid muscle
Source: Medicine (Baltimore). 2024 Jul 26;103(30):e38876. doi: 10.1097/MD.0000000000038876 (PMC11272274; doi:10.1097/MD.0000000000038876)

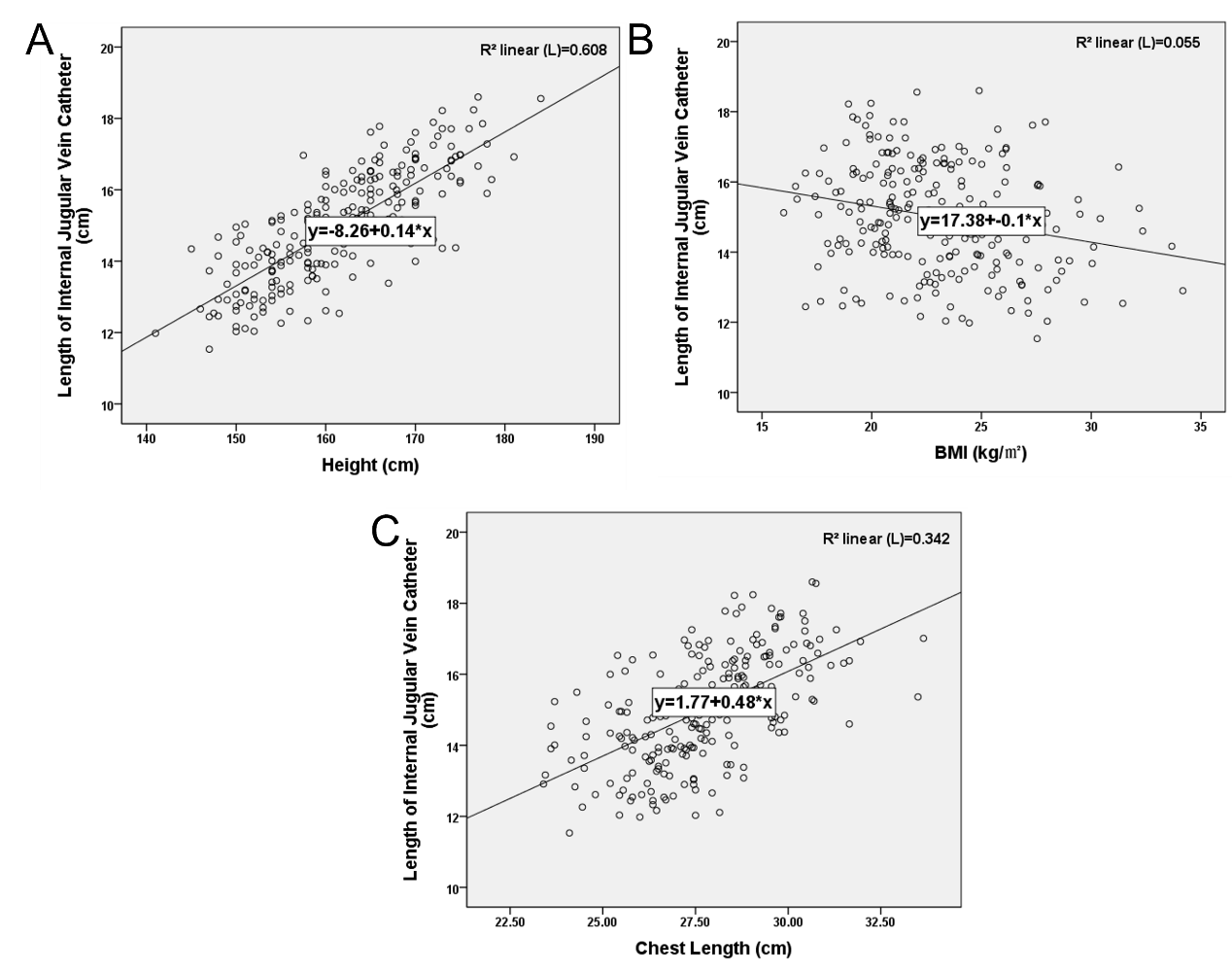

Supplement: Supplementary file 1 [file medi-103-e38876-s001.doc]
